# Bi-Plane Multicolor Scanning Illumination Microscopy with Multispot Excitation and a Distorted Diffraction Grating

**DOI:** 10.3390/bios14110550

**Published:** 2024-11-13

**Authors:** Siwei Li, Yunke Zhang, Zhiwen Liao, Zengyuan Tian, Hairulazwan Hashim, Youjun Zeng, Yandong Zhang

**Affiliations:** 1 College of Mechanical Electronical and Engineering, Zhuhai City Polytechnic, Zhuhai 519000, China; zhcptlsw@163.com (S.L.); yunke@tju.edu.cn (Y.Z.); liaozw_yuy@163.com (Z.L.); tianzengyuan@zhcpt.edu.cn (Z.T.); 2 Department of Electrical Engineering Technology, Universiti Tun Hussein Onn Malaysia, Panchor 84600, Malaysia; azwan@uthm.edu.my; 3 School of Physics & Optoelectronic Engineering, Guangdong University of Technology, Guangzhou 510006, China; zengyoujun@gdut.edu.cn

**Keywords:** super resolution, microscopy, biomedical photonics

## Abstract

Multifocus microscopy has previously been demonstrated to provide volumetric information from a single shot. However, the practical application of this method is challenging due to its weak optical sectioning and limited spatial resolution. Here, we report on the combination of a distorted diffraction grating and multifocal scanning illumination microscopy to improve spatial resolution and contrast. DG is introduced in the emission path of the multifocal scanning illumination microscopy, which splits the fluorescence signal from different sample layers into different diffraction orders. After postprocessing, super-resolution wide-field images of different sample layers can be reconstructed from single 2D scanning.

## 1. Introduction

Fast acquisition of 3D information of specimens remains a major challenge in modern biological microscopy. For most super-resolution microscopy, such as STORM [1,2], SIM [3,4], and STED [5,6], when images of the structure of a sample are made in the focal plane to obtain the complete sample structure information, it is necessary to move the sample or the objective lens axially and image synchronously and then reconstruct a 2D image stack into a 3D sample structure by postprocessing. However, scanning in-depth is a relatively time-consuming method to observe a dynamic biological event, and mechanical moving parts may also introduce an additional drift error. To solve the above problems, the aberration-corrected multifocus microscopy method was proposed [7], which is based on the use of a distorted diffraction grating (DG) to form multiple focus-shifted images. This method can produce an instant focal stack of high-resolution 2D images simultaneously displayed on a single camera, which realizes the simultaneous imaging of multiple sample layers and greatly improves the 3D imaging speed of the system. In 2014, the volumetric PALM/STORM super-resolution method, which relies on MFM, was used to achieve the simultaneous acquisition of nine equally spaced focal planes [8]. In 2017, multifocal SIM was implemented [9], which can improve the volumetric acquisition speed by an order of magnitude. In 2021, the Jörg Enderlein group proposed a rapid multiplane phase-contrast microscopy, which achieved real-time observation of the fast motion of reactivated Chlamydomonas axonemes with sub-µm spatial and 4 ms temporal resolution [10].

However, these 3D microscopy methods are hard to apply to thick tissue samples. Because the power density of the wide-field excitation illumination is weak, it is susceptible to scattering, and it cannot penetrate the cell for 3D imaging. In addition, it is necessary to place customized aberration-corrected optical elements in the detection path to avoid the dispersion effect of the grating, which will significantly increase the cost and application of these methods. To improve the optical sectioning of 3D microscopy, an acoustically driven optofluidic lens integrated in a commercial confocal system can be introduced, which could capture an entire 3D image in a single step. However, the image resolution of the 3D confocal approach is still restricted by light diffraction [11]. Thus, multifocal plane imaging was combined with image scanning microscopy to achieve fast super-resolution 3D imaging [12], but the single-focus excitation mode greatly limited the imaging field of view and speed of the system. In 2023, a new parallelized light-sheet fluorescence microscopy method was developed [13] to achieve simultaneous multiplane imaging, but the results obtained with it depended on 3D deconvolution, and it could not produce images with super resolution.

In addition, in actual cell-sample observations, various tissue structures are often labeled with different dyes, which are used to study the interaction between different structures. To distinguish polychromatic structures, it is usually necessary to introduce additional dichromatic groups and optical components into the detection path. This method is not only limited by the parameters of commercial dichromatic filters but it also has difficulty distinguishing sample structures with similar emission wavelengths. In recent years, some research groups have introduced the method of spectral detection into traditional super-resolution microscopic systems [14,15,16]. These methods divide the detected fluorescence signal into two parts through a spectroscope, where one part is used to obtain the intensity information of the detected sample, and the other part is used to obtain the corresponding spectral information through subsequent optical elements, such as gratings or prisms, by monitoring the intensity distribution of the dispersive fluorescent spots. Although these methods can effectively distinguish different dye-labeling structures in polychromatic samples, they waste a lot of fluorescence information [17,18].

To solve the above problems, bi-plane multicolor scanning illumination microscopy with multispot excitation and a distorted diffraction grating (BMSIM) is proposed in this paper. Based on multifocal scanning illumination microscopy (MSIM) [19], a DG is introduced into the detection during the optical path, so that the fluorescence information of two different sample layers is recorded on the same detection plane at the same time. Next, the super-resolution imaging of the two sample layers is realized by data processing. This hybrid microscopy technique enhances the efficiency of single-scan imaging by a factor of two, halving the time needed to capture a 3D image and potentially minimizing phototoxic side effects. In the meanwhile, on the basis of the dispersion characteristics of the DG, a new image-processing method is proposed, which is capable of effectively distinguishing the structures of samples marked with different dyes, eliminating the need for dichroic mirrors and additional cameras, thereby reducing its hardware requirement.

## 2. Setup and Image Formation

### 2.1. Optical Configuration

**Figure 1:**
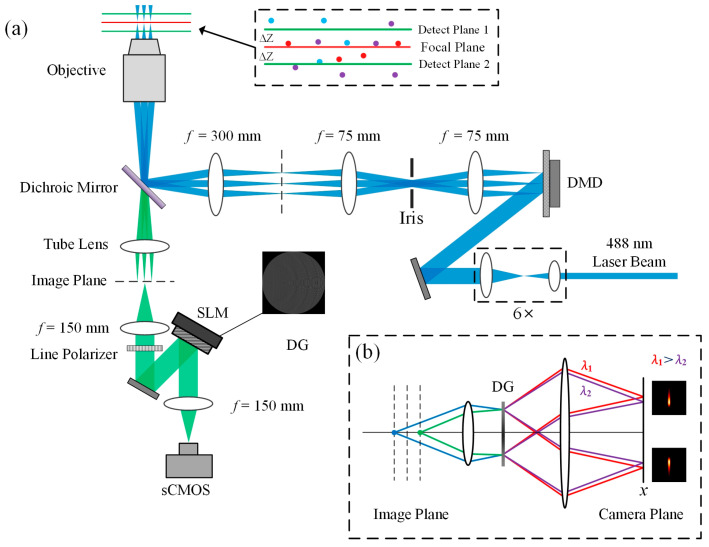
(**a**) Optical configuration of BMSIM. DMD: digital micromirror device, SLM: spatial light modulator*,* DG: distorted diffraction grating; (**b**) imaging principle of the DG in the emission path. Fluorescent signals at different Z-axis positions will emerge at distinct angles after the first lens and DG and then focus at separate locations on the camera plane via the second lens, leading to X-axis broadening of the final focal spots.

The experimental setup of BMSIM is shown in Figure 1. The system utilizes a solid-state laser (Sapphire 488-200 CW CDRH, Coherent, Saxonburg, PA, USA), which provides continuous monochromatic light at a wavelength of 488 nm. A 4*f* configuration constructed from two achromatic lenses is used to expand the excitation beam, and then a digital micromirror device (DMD, DLP 4100, Texas Instruments, Dallas, TX, USA) is illuminated by the collimated beam at 24° off normal, such that an “on”-state micromirror will tilt the output beam to the following system. Subsequently, a 4*f* configuration (*f* = 75 mm) with an optical iris is used to reimage the DMD at the back focal plane of the lens (*f* = 300 mm) and block the undesired diffraction orders of the excitation light generated by the DMD. In the end, each “on”-state pixel of size 10.8 μm ×10.8 μm is demagnified by a factor of 90× by a telescope system consisting of a lens (*f* = 300 mm) and an objective (Nikon, 90×, NA = 1.27) to dimensions of 120 nm × 120 nm in the sample plane. In the emission path, the objective collects the emitted fluorescence from the specimen and guides it into the emission path. Next, a spatial light modulator (SLM, PLUTO, Holoeye, Berlin, Germany) is placed in the Fourier plane of the 4*f* system, and it is optically conjugated to the objective back focal plane. By loading the DG phase map to modulate the fluorescence signal, the signal light emitted by different sample layers is refocused on the different transverse positions of the detection surface, forming two different groups of fluorescence lattices.

**Figure 2:**
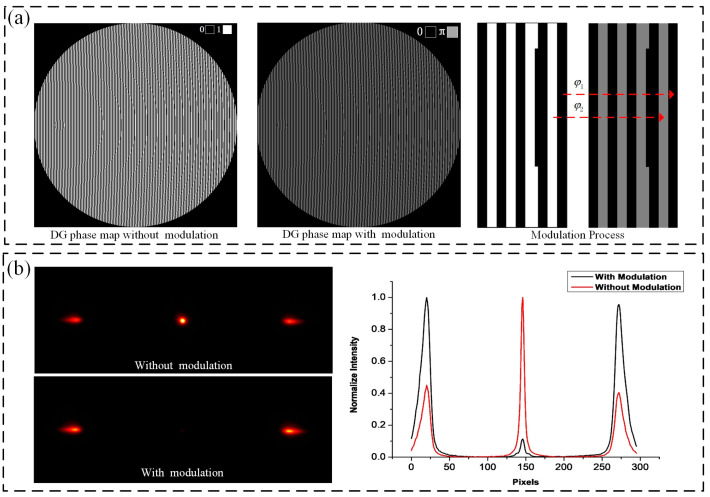
(**a**) The design principles of DG phase map. Conventional DGs feature a binary distribution with light intensity primarily focused on the 0th diffraction order. By modulating the binary grating into a specific phase distribution, the light intensity can be concentrated on a designated diffraction order; (**b**) the fluorescence intensity distributions of DG. The rhodamine sample’s fluorescent image, captured after single-spot illumination, shows light concentrated in the 0th diffraction order with an unmodulated DG. After phase modulation, it shifts to the ±1 orders.

The DG is a specially designed diffraction grating, which introduces an additional quadratic phase factor on the basis of the conventional binary grating design, so that the grating not only maintains the original diffraction splitting ability but also has the lensing effect of a Fresnel zone plate; that is, it has a different lens effect at different diffraction levels. Therefore, when the DG is combined with the lens, objects in different axial positions can be imaged into different lateral positions of the same detection plane [20,21]. The grating used in this experiment was designed based on the method of References [22,23]. Specifically, the approach described in Reference [22] was utilized to generate a binary phase map. Subsequently, the phase distribution within each period of the DG was modulated based on the phase optimization algorithm presented in Reference [23], as shown in Figure 2a. After parameter optimization, the light intensity is concentrated on the diffraction order ±1, and the interval ∆Z of the adjacent diffraction levels is 500 nm, as shown in Figure 2b.

### 2.2. Data Acquisition by BMSIM

**Figure 3:**
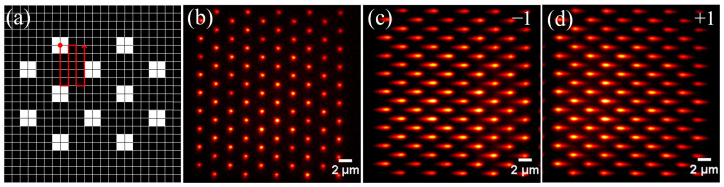
(**a**) DMD pattern. A binary image composed of 0 and 1, where 1-pixel values correspond to the “on” state of the DMD micromirrors, and 0-pixel values correspond to the “off” state; (**b**) rhodamine dye image without the DG phase map; (**c**) rhodamine dye image for diffraction order −1 with the DG; (**d**) rhodamine dye image for diffraction order +1 with the DG.

The DMD can be regarded as a screen with a resolution of 1024 × 768, where each pixel corresponds to a micromirror. The state of each micromirror on the panel is determined by loading a black-and-white image of the same size through control software, with white representing the “on” state and black representing the “off” state. When the displayed image changes, the states of the micromirrors alter correspondingly. To generate multifocus structured illumination, a specific binary pattern (See Figure 3a) is loaded on the DMD, in which each 2 × 2 “on”-state DMD pixel corresponds to one illumination spot, and the horizontal and vertical distances between adjacent spots are 22 pixels and 19 pixels, respectively, which are properly spaced to avoid stacking between focus spots. When starting to scan a sample, the “on” pixel position is changed by switching the pattern of the DMD, and the trace is shown as the red line. The number of scanning steps is 418; each displacement is 1 pixel, which corresponding to the sample plane is 120 nm; the synchronous exposure time of the camera is 8 ms; and the total scanning time is 3 s. It is noteworthy that when the sample exhibits high fluorescence efficiency or when a real grating is utilized, the spacing between illumination points and the camera’s exposure time can be reduced, thereby significantly increasing the efficiency of image acquisition. To observe the actual excitation effect of multifocal structured light, rhodamine dye is used for imaging. When the SLM is in standby, the sample fluorescence signal forms a periodic Gaussian lattice on the detection plane, as shown in Figure 3b. Then, when the DG phase is loaded, the fluorescence signal is affected by the grating and focuses on two different positions of the detection plane, corresponding to the ±1 diffraction orders of the grating, as shown in Figure 3c,d.

### 2.3. System Calibration of BMSIM

**Figure 4:**
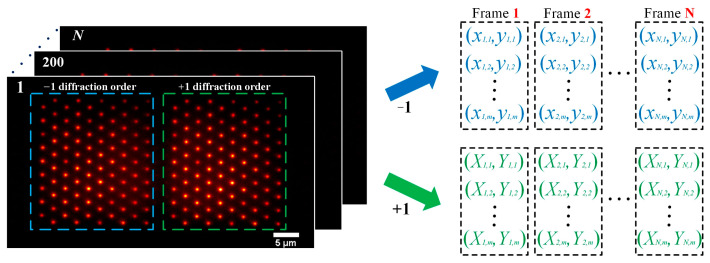
Calibration principle of the excitation spot in BMSIM. By calculating and recording the position of each fluorescent spot (emission wavelength 535 nm) in the image, valuable information can be provided for subsequent image segmentation and spectral calculations.

When postprocessing the data, to improve the computational efficiency, it is necessary to prelabel the position coordinates of each illumination spot. Therefore, a narrowband filter (central wavelength of 535 nm, bandwidth of 10 nm) is placed in front of the detector, and the rhodamine dye sample is scanned. Then, a Gaussian lattice image sequence is synchronously acquired by the detector, as shown in Figure 4. Finally, each excitation center position in the image is located and recorded according to the diffraction order. Among them, the illumination spot coordinates of the two different diffraction orders corresponding to the DMD pattern are recorded, respectively. After the above process, the reference spot calibration of the BMSIM system can be realized.

### 2.4. BMSIM Image-Reconstruction Process

**Figure 5:**
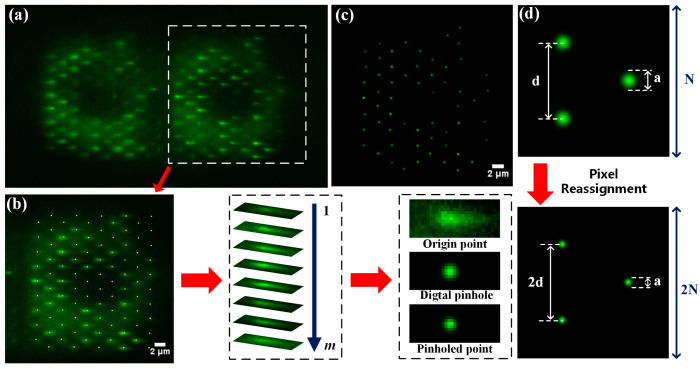
Image reconstruction process of BMSIM. (**a**) Single-image data of BMSIM. The mitochondria form broadened spots in the left and right regions of the camera plane after multifocal illumination; (**b**) processing procedure for single-spot data. The ROI are segmented based on reference coordinate and assigned a digital pinhole, which suppresses background noise while modifying the light intensity to a gaussian distribution; (**c**) spots processed with a digital pinhole. (**d**) Pixel reassignment principle. The implementation involves constructing a doubled-sized new image and replicating the pixel values surrounding each fluorescent point into it, doubling the inter-point distance.

In our study, to obtain super-resolution wide-field images of the sample, we processed the acquired raw images to reconstruct the sample information. The process steps corresponding to diffraction order +1 are as follows: For a single set of raw images, as shown in Figure 5a, the fluorescence spot information in the region of interest (white box) is intercepted to form a subimage, as shown in Figure 5b. Then, the subimage is divided into a series of subareas according to the reference coordinates to ensure that only one spot exists. Next, digital pinholes, which have a Gaussian distribution, are attached sequentially according to the central coordinates, in order to filter out extraneous background fluorescence noise and reshape the spot. After the dispersed spots are recovered to Gaussian spots (see Figure 5c), pixel reassignment [24,25] is used to improve the lateral resolution; the principle behind this is that when a point-scanning microscope system employs a 2D array detector, each pixel can be approximated as a single-point detector with an infinitely small pinhole. When recording the fluorescence information from a sample, each pixel captures a single-point confocal image with a resolution close to the theoretical maximum. By sequentially superimposing these single-pixel images, a super-resolution confocal image with a higher signal-to-noise ratio (SNR) can be generated. However, before superposition, it is necessary to compensate for the lateral shifts between images caused by differences in the pixel positions of the camera. Therefore, pixel reassignment must be introduced, the process and principle of which are illustrated in Figure 5d. Finally, the processed images are sumed and deconvoluted to reconstruct a super-resolution wide-field image.

### 2.5. Principle of Distinguishing Multicolor Samples

**Figure 6:**
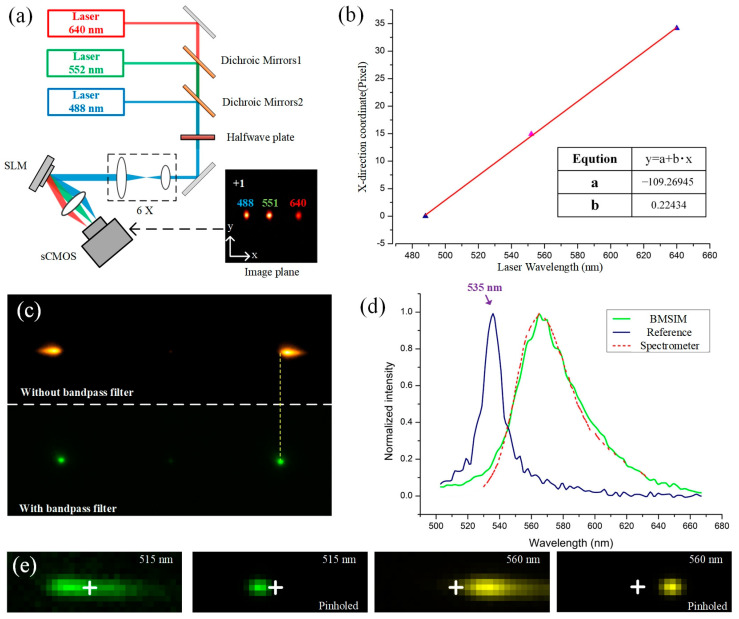
(**a**) Calibration optical setup for the DG dispersion. Multi-wavelength beams are reflected by the SLM and focused on the camera plane with different offsets in the x-direction; (**b**) the calibration curve between pixel position and spectrum. The pixel shift of the focal point exhibits a linear relationship with wavelength; (**c**) image of a rhodamine sample excited by a single spot. When a narrowband filter is placed in the optical path, most wavelengths of the fluorescence signal are blocked, resulting in a focal spot close to a Gaussian distribution; (**d**) comparison of fluorescence spectral detection by BMSIM and spectrometer. The intensity profile of the spot is similar to the spectral curve; (**e**) principle for distinguishing multicolor samples. Estimation of central wavelength via x-direction pixel shift from fluorescence center to reference (535 nm).

In BMSIM, the fluorescence of different wavelengths is influenced by the DG to focus at distinct positions on the detector. To calculate the relationship between wavelength variation and the displacement of the focal spot, three lasers with distinct wavelengths are employed as light sources. After being expanded, the light is projected onto an SLM with the DG phase map. Consequently, the light of different wavelengths reflects at different angles and focuses on the detection plane, resulting in x-direction displacements. The image of the +1 diffraction order is depicted in Figure 6a. Based on the coordinates of the three focal spots and their corresponding wavelengths, a linear fit can be applied to establish the relationship between the wavelengths and the positions, as indicated in Figure 6b.

When the sample is excited by the illumination, the spectrum of the fluorescence spot has a certain width, so that the spot on the detection plane will be broadened in one direction by the grating dispersion, and its profile intensity is consistent with its spectral curve [26], as shown in Figure 6c,d. Therefore, BMSIM can distinguish sample structure with different emission wavelengths based on the spot peak location. Figure 6e demonstrates the distinguishing process of the multicolor sample; when structures of the sample are labeled with different dyes, there will be some differences in the spectral peaks of the excited fluorescence spot, and based on the relative position of the fluorescence signal center of mass and the reference spot (white cross, 535 nm), the fluorescence signal at different wavelengths can be filtered out rapidly and attached by digital pinhole.

## 3. Experimental Results

**Figure 7:**
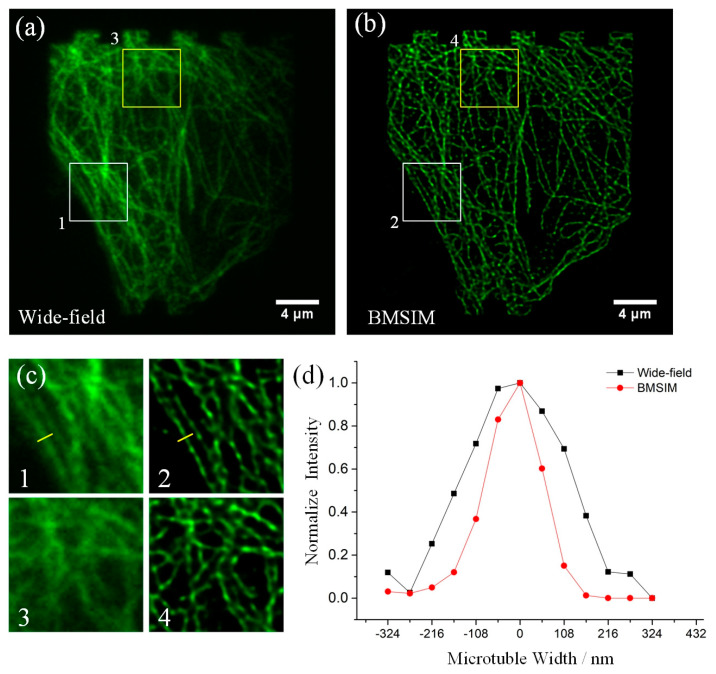
Resolution and contrast enhancement in BMSIM. (**a**) Wide-field image of a microtubule; (**b**) BMSIM image of the same microtubule; (**c**) magnification of the box regions in (**a**,**b**); (**d**) plots of FWHM intensity along the yellow lines in (**c**) for the wide-field image (284 nm) and BMSIM image (155 nm).

To characterize the lateral resolution of BMSIM, bovine pulmonary artery endothelial cells (F14781, Thermo Fisher Scientific, Waltham, MA, USA) were sampled, the results of which are shown in Figure 7. The fluorescence image of microtubules under conventional wide-field illumination are shown in Figure 7a, and Figure 7b illustrate the image reconstructed by BMSIM. By comparing the details of the sample structure in the boxed area (see Figure 7c), the two adjacent microtubules can be clearly observed in the BMSIM image, while in the same area, the dense microtubule fluorescence signals are blurred under wide-field illumination. Finally, the full-width at half maximum (FWHM) intensity of the microtubules corresponding to Figure 7c is shown in Figure 7d, displaying the FWHM of wide-field illumination and BMSIM at 284 nm and 155 nm, respectively. These results indicate that BMSIM achieves a 1.83-fold resolution compared to conventional wide-field microscopy; it is a slightly lower imaging resolution than MSIM due to grating dispersion.

**Figure 8:**
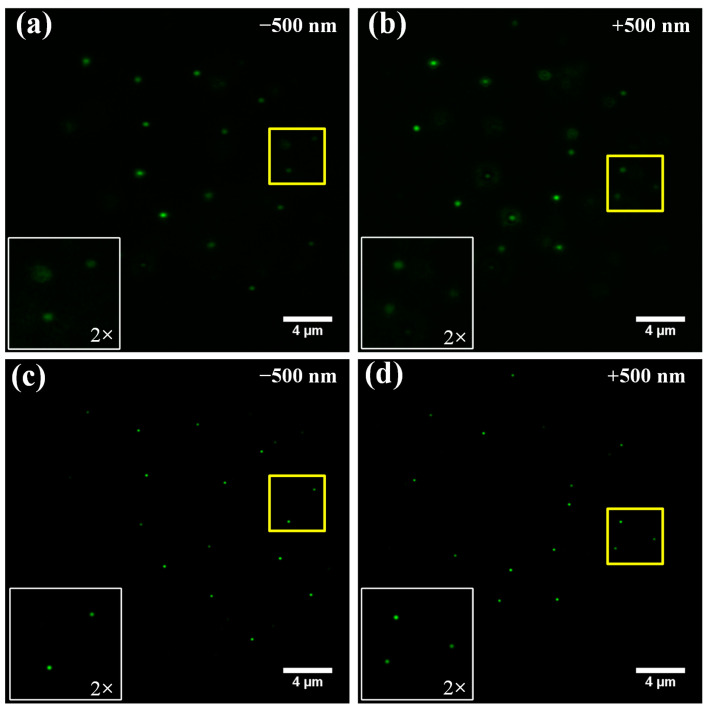
Comparison of imaging results for the fluorescent-bead sample between conventional wide-field microscopy and BMSIM. (**a**,**b**) are the results of wide-field illumination at ± 500 nm depth, respectively; (**c**,**d**) are the results of BMSIM of the sample at ± 500 nm depth, respectively.

To verify the bi-plane imaging capability of BMSIM, a fluorescent-bead solution (ex 488/em 560) was mixed with an agar solution to form a 3D structure sample. Figure 8a,b show images of the fluorescent beads under conventional wide-field illumination with the narrowband filter, and it can be observed that the distributions of fluorescent beads in the two sample layers are significantly different. Figure 8c,d show the reconstructed wide-field image based on BMSIM in which the fluorescent beads have higher resolution and the postprocessing has effectively filtered out the defocused signal, yielding higher optical sectioning.

**Figure 9:**
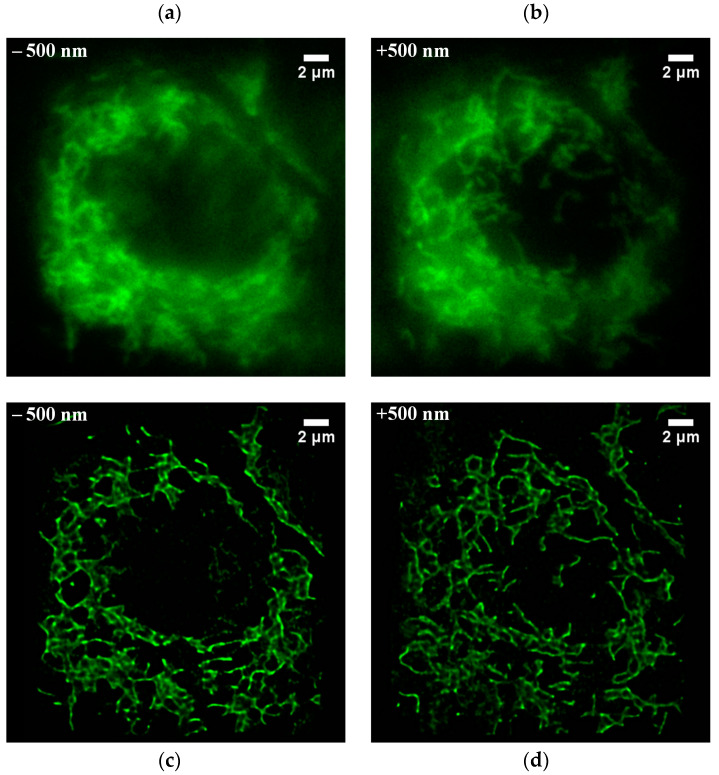
Comparison of imaging results for the mitochondrial sample between conventional wide-field microscopy and BMSIM. (**a**,**b**) are the results of wide-field illumination at ±500 nm depth, respectively. (**c**,**d**) are the results of BMSIM of the sample at ± 500 nm depth, respectively.

Next, to verify BMSIM’s imaging capability further, the mitochondrial structure of live HeLa cells was rapidly scanned, where the structural distribution at axial positions of ±500 nm was reconstructed via postprocessing. Figure 9a,b show wide-field illumination images of the mitochondrial sample at ±500 nm. It can be observed that the conventional illumination mode generates scattering when the thicker sample is excited, which produces a large amount of background fluorescence noise in the dense region of mitochondria, resulting in a loss of structural details and many features being indistinguishable. Figure 9c,d exhibit reconstructed wide-field BMSIM images. With the multispot illumination and the application of the digital pinhole, the background fluorescence signal is effectively suppressed, and a higher section effect is obtained. In addition, the details of the mitochondria are sharper, produced by the pixel reassignment and deconvolution.

**Figure 10:**
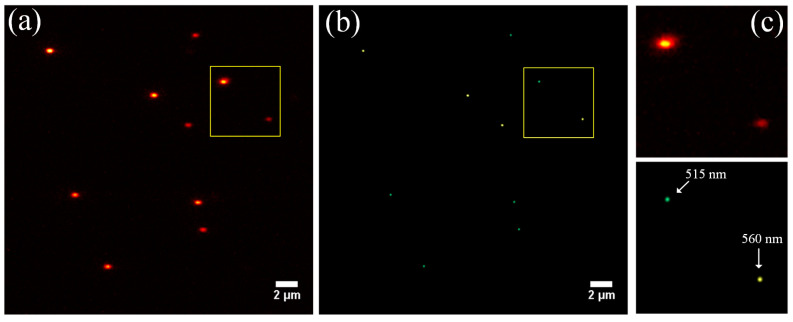
Comparison of imaging results for the dual-color fluorescent-bead sample between conventional wide-field microscopy and BMSIM. (**a**) Wide-field imaging result of fluorescent beads after placing a narrow-band filter in front of the detector; (**b**) multicolor fluorescent-bead imaging result obtained by BMSIM. Beads emitting at different wavelengths have been labeled with corresponding fluorescent colors. (**c**) magnification of the box regions in (**a**,**b**).

Finally, to examine the multicolor imaging capabilities of BMSIM, two fluorescent-bead solutions with different emission wavelengths (ex 505/em 515 and ex 488/em 560) were mixed to form a sample. After that, multicolor wide-field images of the fluorescent beads were obtained by data acquisition, and the resulting image reconstruction is shown in Figure 10. Figure 10a depicts a wide-field illuminated image of fluorescent beads with a narrowband filter, and Figure 10b shows a reconstructed wide-field image produced via BMSIM. Fluorescent beads imaged at two different emission wavelengths, positioned and marked with different colors, can clearly be distinguished. Figure 10c illustrates a magnification of the yellow box areas in Figure 10a,b. By comparison, it can be seen that BMSIM can effectively distinguish the structure of polychromatic samples, which is significant for the study of multicolor labeled biological samples.

## 4. Conclusions and Analysis

In this paper, we proposed a bi-plane multicolor scanning illumination microscopy approach capable of obtaining different sample-layer information from a single 2D scan, called BMSIM. This method introduces a DG into the traditional MSIM system, which allows the fluorescence signals of different sample layers from different positions to be focused into the same detection plane. With this approach, information from the sample layers can be reconstructed via data post-processing. This 3D microscopy method requires less 2D scan time, which makes it faster than conventional MSIM. Thus, the proposed method offers the benefit of potentially lower phototoxic side effects. In addition, the system can be used for simultaneous imaging of multicolor samples via post-processing. Our approach can significantly contribute to the further development of super-resolution microscopy for biological imaging.

## Data Availability

The data that support the findings of this study are available within this manuscript or from the corresponding author on reasonable request.
